# Identifying transient ischemic attack (TIA) patients at high-risk of adverse outcomes: development and validation of an approach using electronic health record data

**DOI:** 10.1186/s12883-022-02776-1

**Published:** 2022-07-12

**Authors:** Laura J. Myers, Anthony J. Perkins, Ying Zhang, Dawn M. Bravata

**Affiliations:** 1 Department of Veterans Affairs (VA) Health Services Research and Development (HSR&D) Precision Monitoring to Transform Care (PRISM) Quality Enhancement Research Initiative (QUERI), Indianapolis, USA; 2 VA HSR&D Center for Health Information and Communication (CHIC), Richard L. Roudebush VA Medical Center, Indianapolis, IN USA; 3 Department of Internal Medicine, Indiana University School of Medicine, Indianapolis, IN USA; 4 Regenstrief Institute, Indianapolis, IN USA; 5 Department of Biostatistics and Health Data Science, Indiana University School of Medicine, Indianapolis, IN USA; 6 Department of Biostatistics, College of Public Health, University of Nebraska Medical Center, Omaha, NE USA; 7 Department of Neurology, Indiana University School of Medicine, Indianapolis, IN USA

**Keywords:** Cerebrovascular disease, Transient ischemic attack, Risk stratification, Outcomes

## Abstract

**Background:**

Risk-stratification tools that have been developed to identify transient ischemic attack (TIA) patients at risk of recurrent vascular events typically include factors which are not readily available in electronic health record systems. Our objective was to evaluate two TIA risk stratification approaches using electronic health record data.

**Methods:**

Patients with TIA who were cared for in Department of Veterans Affairs hospitals (October 2015—September 2018) were included. The six outcomes were mortality, recurrent ischemic stroke, and the combined endpoint of stroke or death at 90-days and 1-year post-index TIA event. The cohort was split into development and validation samples. We examined the risk stratification of two scores constructed using electronic health record data. The Clinical Assessment Needs (CAN) score is a validated measure of risk of hospitalization or death. The PREVENT score was developed specifically for TIA risk stratification.

**Results:**

A total of *N* = 5250 TIA patients were included in the derivation sample and *N* = 4248 in the validation sample. The PREVENT score had higher c-statistics than the CAN score across all outcomes in both samples. Within the validation sample the c-statistics for the PREVENT score were: 0.847 for 90-day mortality, 0.814 for 1-year mortality, 0.665 for 90-day stroke, and 0.653 for 1-year stroke, 0.699 for 90-day stroke or death, and 0.744 for 1-year stroke or death. The PREVENT score classified patients into categories with extreme nadir and zenith outcome rates. The observed 1-year mortality rate among validation patients was 7.1%; the PREVENT score lowest decile of patients had 0% mortality and the highest decile group had 30.4% mortality.

**Conclusions:**

The PREVENT score had strong c-statistics for the mortality outcomes and classified patients into distinct risk categories. Learning healthcare systems could implement TIA risk stratification tools within electronic health records to support ongoing quality improvement.

**Registration:**

ClinicalTrials.gov Identifier: NCT02769338.

## Introduction

Patients with transient ischemic attack (TIA) are at risk of recurrent vascular events [1–3]. Several risk stratification tools have been developed to identify TIA patients with the greatest risk of recurrent vascular events [4]. The ABCD_2_ score is the most commonly used TIA risk stratification tool [4]. The ABCD_2_ score includes age, blood pressure, clinical features (e.g., weakness or speech impairment), neurologic symptom duration, and diabetes [5].

Although the ABCD_2_ can be calculated prospectively by clinicians caring for patients, electronic health record systems typically do not include neurological symptom descriptions as data fields; therefore, this score, and others that include results from brain imaging cannot be constructed from electronic health record data [4]. TIA risk stratification tools that effectively distinguish low- from high-risk patients and that can be deployed within electronic health record systems are needed to support the Learning Healthcare System model which involves learning from data to improve practice. For example, Learning Healthcare Systems need robust risk adjustment models to identify facilities that have outlier status (those with either better-than-expected or worse-than-expected outcome rates). By exploring differences in policies, practices, infrastructure, and culture between high-performing and low-performing facilities opportunities for quality improvement may be recognized. The objective of this project was to evaluate two TIA risk stratification approaches based on electronic health record data.

## Methods

### Cohort

A sample from the cohort that was constructed for the Protocol-Guided Rapid Evaluation of Veterans Experiencing New Transient Neurological Symptoms (PREVENT; clinicaltrials.gov: NCT02769338) study was used for this project [6, 7]. We identified patients with TIA who were cared for in any Department of Veterans Affairs (VA) Emergency Department (ED) or inpatient setting based on primary discharge codes for TIA from October 1, 2015 to September 30, 2018 (*International Classification of Disease* ICD-10 G45.0, G45.1, G45.8, G45.9, I67.848 ) [8]. The first TIA event during the study period per patient was included. This project received human subjects (institutional review board [IRB]) and VA research and development committee approvals. The institutional review board waived the need for patient consent.

### Outcomes

We examined three outcomes at 90-days and 1-year post-index TIA. The all-cause mortality rate (defined as death from any cause within 90-days or 1-year of presentation for the index event) was obtained from the VA Vital Status File [9]. Recurrent ischemic stroke (defined as an ischemic stroke in the ED or inpatient stay within 90-days or 1-year of discharge of the index TIA event) was identified using primary diagnosis codes in the ED or inpatient setting using a combination of both VA and fee-basis data (which describes healthcare in non-VA facilities that was paid for by the VA). Therefore, recurrent strokes which occurred in community hospitals, but which were not paid for by the VA, were not included. The combined endpoint of stroke or death was also evaluated at 90-days and 1-year post-index TIA.

### Data sources

Data were obtained from the VA Corporate Data Warehouse (CDW) [10] which includes: inpatient and outpatient data files (e.g., diagnostic and procedure codes) in the five-years pre-event to identify past medical history, [11] healthcare utilization, receipt of procedures (Current Procedural Terminology [CPT], Healthcare Common Procedures Coding System [HCPCS], and *ICD*-9 and *ICD*-10 procedure codes), vital signs, laboratory data, orders, medications and clinical consults. Fee-Basis Data were also used to identify inpatient and outpatient healthcare utilization and medical history.

The Clinical Assessment Needs (CAN) version 2.0 score is a validated measure of risk of hospitalization, death, or the combination of hospitalization or death within 90-days or 1-year that is calculated for Veterans in VA healthcare [12]. The CAN score version 2.0 is updated weekly for patients that are assigned to a primary care Patient Aligned Care Team (PACT), are Veterans, not hospitalized on the date the score is run, and are alive as of the date the score is generated. The CAN score is based on 32–36 data elements obtained from the CDW including: sociodemographics, healthcare utilization (e.g., clinic visits, inpatient admissions, ED and urgent care visits), vital signs, medications, laboratory data, and number and type of comorbidities.

### Analysis

The original sample consisted of 8270 patients from Oct 1, 2015 to March 31, 2018. The cohort was randomly split into training (*n* = 5506) and validation samples (*n* = 2764 ) [13]. Within the training sample, logistic regression models were used to identify the patient characteristics that were associated with the outcomes [14]. Separate risk adjustment models were constructed for each outcome. For the purpose of this analysis, we added all TIA patients from April 1, 2018 to September 30, 2018 to the validation sample and excluded all patients with missing CAN scores resulting in a final sample size of 5250 for the training sample and 4248 for the validation sample. The final models that were constructed on the training set (referred to as the PREVENT scores) were then applied to the validation set. Chi-square tests, t-tests, or Wilcoxon Rank Sum tests were used to compare whether patient characteristics differed between the development and validation samples. C-statistics (area under the receiver operating characteristic [ROC] curve) [4] and observed (unadjusted) outcome rates across risk categories (including identification of patients with extremely low [nadir] or high [zenith] outcome rates) [15] were used to evaluate the performance of the final risk model. All analyses were performed using SAS Enterprise Guide version 7.11.

### Data sharing statement

The dataset supporting the conclusions of this article is not available. According to Department of Veterans Affairs (VA) policy, these data are stored behind the VA firewall and cannot be shared even after deidentification. Investigators interested in analyses of the existing data are encouraged to contact the corresponding author.

## Results

A total of *N* = 5250 TIA patients were included in the derivation sample and *N* = 4248 in the validation sample; their baseline characteristics are provided in Table 1.

**Table 1:** Comparison of baseline characteristics between derivation and validation samples

	Derivation Sample (*N* = 5250)	Validation Sample (*N* = 4248)	***P***-Value
**Patient Characteristics**			
**Index Event**			
Index Event			0.404
Emergency Department-Only	32.4 (1702)	31.6 (1343)	
Admitted	67.6 (3548)	68.4 (2905)	
Weekday presentation	79.9 (4196)	79.8 (3388)	0.839
Left Against Medical Advice (AMA)	4.5 (235)	5.0 (214)	0.200
**Demographics**			
Age (years): mean ± standard deviation	70.41 (11.27)	70.46 (11.31)	0.811
Median (IQR)*	70.0 (64.0–78.0)	70.0 (64.0–78.0)	0.958
Male Gender	94.9 (4983)	94.8 (4028)	0.838
Race			0.960
White	75.7 (3972)	75.8 (3221)	
Black	19.7 (1034)	19.7 (837)	
Asian	0.5 (26)	0.6 (24)	
Other	0.7 (36)	0.6 (26)	
Unknown	3.5 (182)	3.3 (140)	
Hispanic ethnicity	7.2 (380)	7.3 (311)	
**Past Medical History**			
Transient Ischemic Attack (TIA) in prior 30 days	4.4 (230)	3.6 (155)	0.072
Stroke in prior 30 days	5.8 (302)	5.7 (243)	0.947
Diabetes mellitus	42.4 (2226)	43.1 (1832)	0.477
Atrial fibrillation	16.8 (883)	17.0 (724)	0.772
Myocardial infarction	6.9 (362)	7.7 (328)	0.123
Congestive heart failure	15.0 (789)	15.4 (655)	0.598
Carotid endarterectomy or stent	1.0 (50)	0.8 (36)	0.591
Chronic obstructive pulmonary disease (COPD)	21.8 (1147)	22.2 (942)	0.702
Peripheral arterial disease	14.2 (745)	14.7 (625)	0.471
Dementia	8.0 (418)	7.3 (309)	0.210
Chronic kidney disease	17.4 (916)	18.4 (781)	0.236
**Past Medical History**			
Dialysis	1.5 (78)	1.3 (57)	0.556
Cancer	11.3 (592)	11.7 (499)	0.475
Hypertension	75.8 (3982)	77.5 (3292)	0.059
Hyperlipidemia	61.3 (3218)	63.0 (2678)	0.081
Speech deficit	4.6 (239)	5.1 (217)	0.208
Motor deficit, hemiplegia	14.6 (766)	16.3 (692)	0.022
Sleep apnea	19.3 (1015)	19.1 (812)	0.788
Alcohol dependence	7.4 (388)	8.2 (350)	0.125
Depression	22.9 (1200)	23.8 (1012)	0.268
History of venous thromboembolism (deep vein thrombosis or pulmonary embolism)	3.4 (177)	3.2 (136)	0.645
Intracranial hemorrhage	5.0 (263)	5.4 (229)	0.405
Gastrointestinal bleeding	0.7 (37)	0.6 (26)	0.580
Migraine	3.2 (169)	3.6 (151)	0.368
Medications prior to index event			
Antihypertensives	85.6 (4493)	86.0 (3653)	0.567
Statin	80.9 (4247)	82.6 (3509)	0.032
Aspirin	75.0 (3940)	74.7 (3173)	0.693
Warfarin	11.7 (613)	10.3 (439)	0.038
Comorbidity			
CHA2DS2−VASc*	3.21 (1.45)	3.21 (1.43)	0.937
HASBLED*	2.22 (1.06)	2.22 (1.04)	0.993
Charlson: mean ± standard deviation	2.83 (2.64)	2.92 (2.70)	0.081
Median (IQR)	2.0 (1.0–4.0)	2.0 (1.0–4.0)	0.111
Smoker	28.4 (1493)	29.5 (1253)	0.258
Palliative care, hospice	3.0 (160)	2.6 (112)	0.232
**Present on Admission**			
Concomitant Myocardial Infarction (MI)	2.0 (105)	2.4 (101)	0.209
Concomitant Congestive Heart Failure (CHF)	1.9 (100)	1.7 (74)	0.556
**Laboratory and Vital Signs**			
APACHE:* mean ± standard deviation	9.83 (6.75)	10.14 (6.70)	0.026
Median (IQR)	9.0 (4.0–14.0)	9.0 (5.0–14.0)	0.007
First Systolic blood pressure (mm Hg):			
Mean Systolic (SD)	147.41 (25.38)	146.78 (25.05)	0.226
Median Systolic (IQR)	146.0 (130.0–164.0)	146.0 (130.0–163.0)	0.320
First Diastolic blood pressure (mm Hg):			
Mean Diastolic (SD)	81.25 (14.20)	81.31 (14.09)	0.846
Median Diastolic (IQR)	80.0 (72.0–90.0)	81.0 (72.0–90.0)	0.721
Average Systolic blood pressure 90 days post discharge (mm Hg):			
Mean Systolic (SD)	131.10 (15.35)	130.59 (15.63)	0.144
Median Systolic (IQR)	131.0 (121.0–140.0)	130.0 (120.0–139.5)	0.132
Average Diastolic blood pressure 90 days post discharge (mm Hg):			
Mean Diastolic (SD)	74.42 (9.84)	74.21 (9.78)	0.334
Median Diastolic (IQR)	74.6 (68.0–81.0)	74.0 (68.0–80.5)	0.309
**Outcomes**			
Death 90-days	2.2 (116)	2.1 (88)	0.645
Death 1-year	7.7 (405)	7.1 (303)	0.283
Stroke 90-days	3.2 (164)	3.2 (134)	0.943
Stroke 1-year	5.3 (257)	5.6 (224)	0.444
Stroke or Death 90-days	5.2 (272)	5.1 (215)	0.793
Stroke or Death 1-year	12.0 (629)	11.8 (500)	0.752

*IQR refers to interquartile range; the CHA_2_DS_2−_VASc score is a measure of thromboembolic risk among patients with atrial fibrillation; the HASBLED score is a measure of risk of major bleeding; and the modified APACHE III score is a measure of physiological disease severity

The two samples were similar with the following exceptions: more validation patients had hemiplegia (16.3% versus 14.6%, *p* = 0.022); more validation patients were taking statins prior to the index-TIA (82.6% versus 80.9%, *p* = 0.032); fewer validation patients were taking warfarin prior to the index-TIA (10.3% versus 11.7%, *p* = 0.038); and validation patients had higher APACHE scores indicating modestly greater physiologic disease severity (mean 10.14 ± standard deviation 6.70 versus 9.83 ± 6.75; *p* = 0.026). The overall outcome rates in the validation set (Table 2) were: 2.1% 90-day mortality, 7.1% 1-year mortality, 3.2% 90-day stroke, 5.6% 1-year stroke, 5.1% 90-day stroke or death, and 11.8% 1-year stroke or death. The factors that were included in the final PREVENT score are provided in Table 3.

**Table 2:** Comparison of the CAN Score versus PREVENT Score

Outcome	Training Sample (*N* = 5250)		Validation Sample (*N* = 4248)	
	CAN Score	PREVENT Score	CAN Score	PREVENT Score
	c-statistic	c-statistic	c-statistic	c-statistic
**Mortality**				
90-Days	0.742	0.825	0.752	0.847
1-Year	0.753	0.810	0.745	0.814
**Stroke**				
90-Days	0.560	0.729	0.611	0.665
1-Year	0.601	0.721	0.634	0.653
**Stroke or Mortality**				
90-Days	0.614	0.721	0.651	0.699
1-Year	0.669	0.733	0.691	0.744

**Table 3:** PREVENT risk models for each outcome

Risk Model for 1-Year Mortality	Parameter Estimate	Standard Error	OR (95% CI)	P-value
Intercept	−7.79	0.53		
Age	0.06	0.01	1.064 (1.052, 1.077)	< 0.001
Female	−0.10	0.33	0.901 (0.470, 1.726)	0.754
Black race	−0.28	0.16	0.758 (0.557, 1.033)	0.079
Other Race	0.11	0.26	1.119 (0.668, 1.875)	0.670
Charlson Comorbidity Index	0.18	0.02	1.195 (1.146, 1.246)	< 0.001
Congestive Heart Failure (BNP* > 200 ng/ml)	0.75	0.26	2.111 (1.273, 3.501)	0.004
Current Smoker	0.41	0.13	1.505 (1.165, 1.945)	0.002
History of Intracranial Hemorrhage	0.46	0.21	1.585 (1.052, 2.387)	0.028
History of Atrial Fibrillation	0.36	0.13	1.437 (1.122, 1.840)	0.004
History of Cirrhosis	0.98	0.30	2.658 (1.477, 4.784)	0.001
History of Dementia	0.74	0.14	2.092 (1.578, 2.774)	< 0.001
History of Dialysis	0.87	0.30	2.388 (1.334, 4.275)	0.003
History of Diabetes	−0.38	0.13	0.683 (0.532, 0.877)	0.003
History of Hyperlipidemia	−0.39	0.12	0.674 (0.538, 0.846)	0.001
Non-steroidal anti-inflammatory drug (NSAID)	−0.42	0.16	0.654 (0.474, 0.902)	0.010
Hospice/Palliative Care	0.89	0.19	2.433 (1.661, 3.564)	< 0.001
Syncope	−0.31	0.13	0.737 (0.576, 0.942)	0.015
Systolic Blood Pressure (mmHg)				
Missing	0.89	0.44	2.438 (1.024, 5.807)	0.044
< 110	0.60	0.26	1.819 (1.092, 3.029)	0.022
110–139	0.30	0.21	1.343 (0.893, 2.022)	0.157
140–159	0.06	0.21	1.062 (0.698, 1.616)	0.778
160–179	0.16	0.23	1.179 (0.758, 1.835)	0.465
180+ (reference)			1.000	
**Risk Model for 1-Year Mortality or Stroke**				
Intercept	−5.20	0.38		
Age	0.03	0.00	1.030 (1.021, 1.039)	< 0.001
Female	0.02	0.23	1.023 (0.648, 1.615)	0.922
Black race	−0.38	0.12	0.687 (0.540, 0.873)	0.002
Other Race	0.12	0.20	1.127 (0.763, 1.666)	0.548
APACHE^†^ score	0.02	0.01	1.020 (1.007, 1.033)	0.002
Charlson Comorbidity Index	0.09	0.02	1.097 (1.060, 1.136)	< 0.001
Current Smoker	0.36	0.10	1.429 (1.173, 1.741)	< 0.001
Hemiplegia	0.34	0.11	1.404 (1.122, 1.755)	0.003
History of Intracranial Hemorrhage	0.55	0.17	1.734 (1.249, 2.408)	0.001
History of Congestive Heart Failure	0.29	0.11	1.336 (1.071, 1.666)	0.010
History of Cirrhosis	0.59	0.27	1.796 (1.055, 3.057)	0.031
History of Dementia	0.52	0.13	1.688 (1.306, 2.183)	< 0.001
History of Dialysis	0.49	0.27	1.634 (0.957, 2.790)	0.072
History of Hyperlipidemia	−0.35	0.09	0.705 (0.587, 0.847)	< 0.001
History of Stroke	0.47	0.11	1.596 (1.277, 1.994)	< 0.001
Hospice/Palliative Care	0.78	0.18	2.175 (1.530, 3.093)	< 0.001
Non-steroidal anti-inflammatory drug (NSAID)	−0.28	0.12	0.758 (0.601, 0.956)	0.019
Anti-hypertensive medication	0.48	0.16	1.613 (1.188, 2.188)	0.002
**Risk Model for 90-Day Mortality**				
Intercept	−8.01	0.84		
Age	0.04	0.01	1.043 (1.023, 1.064)	< 0.001
Female	0.06	0.54	1.061 (0.366, 3.079)	0.913
Black race	−0.15	0.27	0.859 (0.507, 1.455)	0.572
Other race	−0.54	0.58	0.582 (0.188, 1.807)	0.349
Charlson Comorbidity Index	0.19	0.03	1.206 (1.136, 1.281)	< 0.001
Congestive Heart Failure (BNP > 200 ng/ml)	0.85	0.37	2.331 (1.128, 4.814)	0.022
Hemiplegia	0.59	0.23	1.799 (1.157, 2.796)	0.009
History of Atrial Fibrillation	0.57	0.21	1.760 (1.168, 2.652)	0.007
Hospice/Palliative Care	1.72	0.25	5.592 (3.409, 9.171)	< 0.001
Syncope	−0.79	0.24	0.453 (0.282, 0.726)	0.001
Systolic Blood Pressure (mmHg)				
Missing	1.81	0.59	6.117 (1.943, 19.258)	0.002
< 110	0.25	0.46	1.279 (0.522, 3.133)	0.591
110–139	0.37	0.36	1.451 (0.710, 2.963)	0.308
140–159	−0.19	0.39	0.829 (0.386, 1.781)	0.630
160–179	−0.12	0.42	0.884 (0.391, 2.000)	0.768
180+ (reference)			1.000	
**Risk Model for 90-Day Mortality or Stroke**				
Intercept	−3.51	0.51		
Age	0.004	0.01	1.004 (0.991, 1.016)	0.569
Female	0.05	0.32	1.050 (0.565, 1.953)	0.877
Black race	−0.50	0.18	0.606 (0.428, 0.860)	0.005
Other Race	−0.03	0.29	0.975 (0.548, 1.733)	0.931
APACHE score	0.02	0.01	1.024 (1.006, 1.043)	0.011
Charlson Comorbidity Index	0.12	0.02	1.127 (1.078, 1.179)	< 0.001
Current Smoker	0.33	0.14	1.385 (1.052, 1.822)	0.020
Hemiplegia	0.63	0.15	1.876 (1.391, 2.530)	< 0.001
History of Intracranial Hemorrhage	0.58	0.22	1.780 (1.146, 2.764)	0.010
History of Depression	−0.42	0.16	0.656 (0.475, 0.905)	0.010
History of Stroke	0.42	0.16	1.526 (1.125, 2.072)	0.007
History of Transient Ischemic Attack	−0.29	0.13	0.748 (0.581, 0.964)	0.025
Hospice/Palliative Care	1.22	0.22	3.373 (2.203, 5.165)	< 0.001
Systolic Blood Pressure (mmHg)				
Missing	0.86	0.42	2.359 (1.032, 5.389)	0.042
< 110	−0.71	0.30	0.491 (0.274, 0.882)	0.017
110–139	−0.36	0.20	0.700 (0.470, 1.043)	0.080
140–159	−0.66	0.21	0.516 (0.341, 0.783)	0.002
160–179	−0.31	0.22	0.732 (0.480, 1.117)	0.148
180+ (reference)			1.000	
**Risk Model for 1-Year Stroke**				
Intercept	−2.51	0.51		
Age	−0.01	0.01	0.988 (0.976, 1.000)	0.057
Female	−0.03	0.30	0.971 (0.535, 1.762)	0.923
African-American race	−0.24	0.17	0.784 (0.562, 1.094)	0.152
Other Race	0.00	0.28	1.001 (0.575, 1.743)	0.996
Hemiplegia	0.64	0.15	1.897 (1.405, 2.561)	< 0.001
History of Intracranial Hemorrhage	0.59	0.23	1.812 (1.145, 2.869)	0.011
History of Cancer	−0.47	0.26	0.628 (0.376, 1.048)	0.075
History of Diabetes	0.43	0.13	1.538 (1.188, 1.990)	0.001
History of Liver Disease	−0.78	0.37	0.457 (0.220, 0.948)	0.036
History of Stroke	1.06	0.15	2.883 (2.167, 3.836)	< 0.001
History of Transient Ischemic Attack	−0.34	0.13	0.710 (0.547, 0.921)	0.010
Antihypertensive Medication	0.77	0.26	2.166 (1.309, 3.585)	0.003
Systolic Blood Pressure (mmHg)				
Missing	−0.04	0.52	0.959 (0.348, 2.641)	0.935
< 110	−0.67	0.32	0.513 (0.274, 0.962)	0.038
110–139	−0.81	0.20	0.446 (0.301, 0.662)	< 0.001
140–159	−0.63	0.20	0.534 (0.363, 0.786)	0.001
160–179	−0.29	0.20	0.745 (0.500, 1.110)	0.148
180+ (reference)			1.000	
**Risk Model for 90-Day Stroke**				
Intercept	−1.50	0.56		
Age	−0.02	0.01	0.978 (0.964, 0.993)	0.003
Female	−0.11	0.38	0.897 (0.423, 1.899)	0.776
African-American race	−0.50	0.22	0.607 (0.392, 0.941)	0.026
Other Race	0.03	0.34	1.032 (0.529, 2.013)	0.926
Hemiplegia	0.68	0.19	1.979 (1.368, 2.863)	0.000
History of Intracranial Hemorrhage	0.65	0.28	1.923 (1.116, 3.313)	0.019
History of Diabetes	0.49	0.16	1.636 (1.193, 2.243)	0.002
History of Peripheral Vascular Disease	0.56	0.20	1.748 (1.181, 2.587)	0.005
History of Stroke	0.87	0.18	2.378 (1.663, 3.401)	< 0.001
History of Transient Ischemic Attack	−0.50	0.16	0.605 (0.440, 0.832)	0.002
Systolic Blood Pressure (mmHg)				
Missing	0.21	0.52	1.229 (0.442, 3.416)	0.693
< 110	−1.46	0.46	0.232 (0.095, 0.567)	0.001
110–139	−0.98	0.24	0.373 (0.235, 0.594)	< 0.001
140–159	−0.92	0.24	0.400 (0.251, 0.638)	< 0.001
160–179	−0.48	0.24	0.618 (0.384, 0.993)	0.047
180+ (reference)			1.000	

*BNP refers to B-type natriuretic peptide

^†^The APACHE score is a measure of physiological disease severity

Both the CAN score and the PREVENT score had higher c-statistics for the mortality outcomes than the recurrent stroke or combined stroke or death outcomes and both scores performed similarly in the development and the validation sets (Table 2). The PREVENT score had higher c-statistics than the CAN score across all outcomes in both the development and validation samples (Table 2). Within the validation sample the c-statistics for the PREVENT score were: 0.847 for 90-day mortality, 0.814 for 1-year mortality, 0.665 for 90-day stroke, and 0.653 for 1-year stroke, 0.699 for 90-day stroke or death, and 0.744 for 1-year stroke or death.

**Fig. 1:**
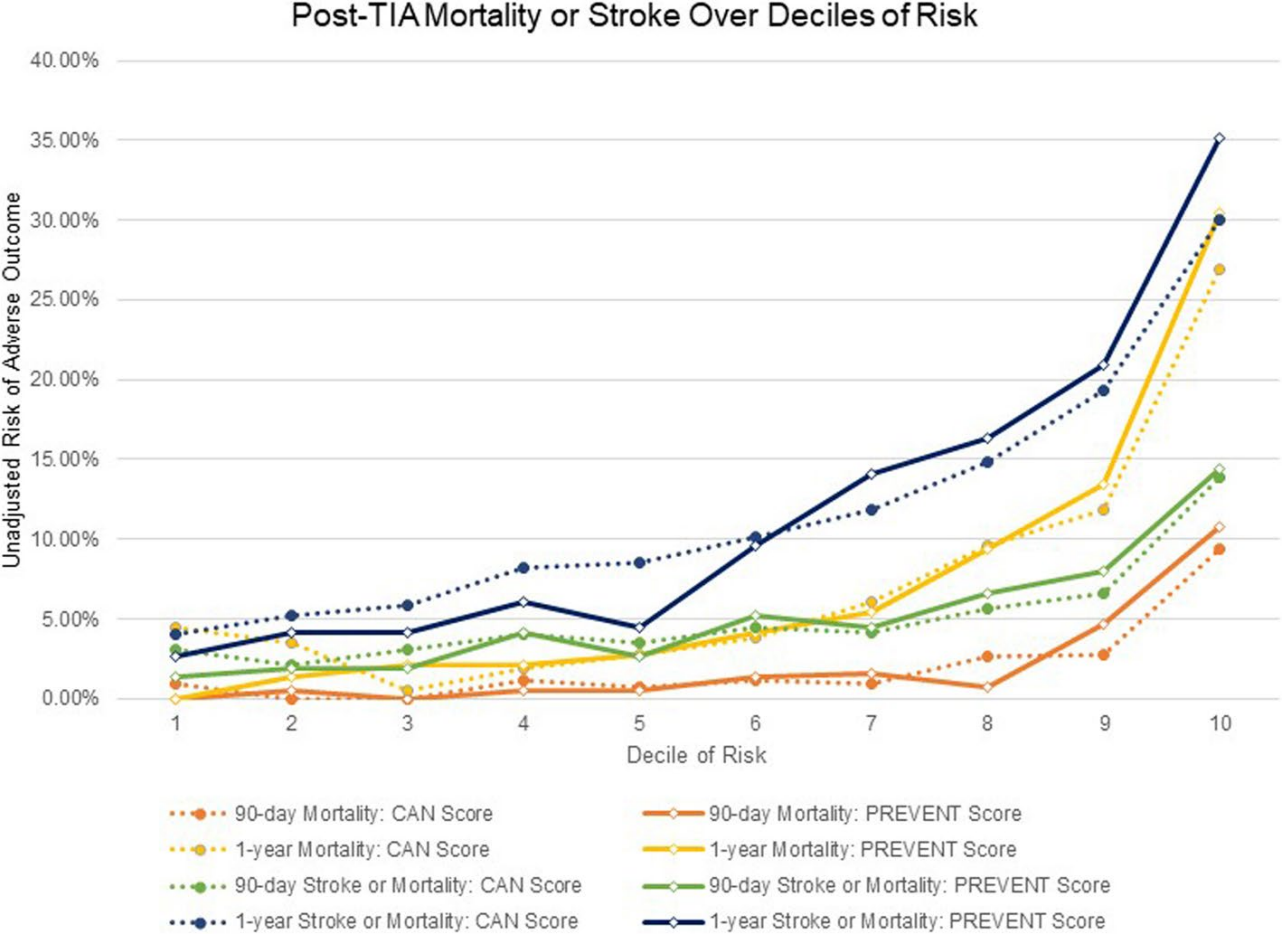
Observed outcome rates: CAN score and PREVENT score

Both scores distinguished low-risk from high-risk patients, however the PREVENT score classified patients into categories with more extreme nadir and zenith outcome rates (Table 4; Fig. 1). For example, as described above, the observed 1-year mortality rate among the patients in the validation sample was 7.1%. The PREVENT score, when split into deciles created a nadir group of patients with 0% 1-year mortality and a zenith group with 30.4% 1-year mortality.

**Table 4:** Comparison of unadjusted outcomes by risk deciles for the validation sample

Outcome	Overall	Risk Deciles									
	N (%)	1	2	3	4	5	6	7	8	9	10
**90-day Mortality**	88 (2.1)										
CAN Score		4 (0.9)	4 (0.0)	0 (0.0)	5 (1.2)	3 (0.7)	5 (1.2)	4 (0.9)	11 (2.6)	12 (2.8)	40 (9.4)
PREVENT Score		0 (0.0)	2 (0.5)	0 (0.0)	2 (0.5)	2 (0.5)	6 (1.4)	7 (1.6)	3 (0.7)	20 (4.7)	46 (10.8)
**1-year Mortality**	303 (7.1)										
CAN Score		19 (4.5)	15 (3.5)	2 (0.5)	8 (1.9)	12 (2.8)	16 (3.8)	26 (6.1)	41 (9.6)	50 (11.8)	114 (26.9)
PREVENT Score		0 (0.0)	6 (1.4)	9 (2.1)	9 (2.1)	12 (2.8)	18 (4.2)	23 (5.4)	40 (9.4)	57 (13.4)	129 (30.4)
**90-day Stroke**	134 (3.2)										
CAN Score		8 (1.9)	8 (1.9)	10 (2.4)	9 (2.2)	15 (3.6)	12 (2.9)	9 (2.2)	17 (4.1)	19 (4.6)	27 (6.5)
PREVENT Score		6 (1.4)	7 (1.7)	6 (1.4)	6 (1.4)	11 (2.6)	12 (2.9)	12 (2.9)	20 (4.8)	26 (6.2)	28 (6.7)
**1-year Stroke**	224 (5.6)										
CAN Score		12 (3.0)	12 (3.0)	10 (2.5)	21 (5.3)	20 (5.0)	21 (5.3)	18 (4.5)	30 (7.6)	37 (9.3)	43 (10.8)
PREVENT Score		16 (4.0)	9 (2.3)	14 (3.5)	16 (4.0)	15 (3.8)	13 (3.2)	21 (5.3)	28 (7.0)	36 (9.1)	56 (14.1)
**90-day Stroke or Mortality**	215 (5.1)										
CAN Score		13 (3.1)	9 (2.1)	13 (3.1)	17 (4.0)	15 (3.5)	19 (4.5)	18 (4.2)	24 (5.6)	28 (6.6)	59 (13.9)
PREVENT Score		6 (1.4)	8 (1.9)	8 (1.9)	18 (4.2)	11 (2.6)	22 (5.2)	19 (4.5)	28 (6.6)	34 (8.0)	61 (14.4)
**1-year Stroke or Mortality**	500 (11.8)										
CAN Score		17 (4.0)	22 (5.2)	25 (5.9)	35 (8.2)	36 (8.5)	43 (10.1)	50 (11.8)	63 (14.8)	82 (19.3)	127 (30.0)
PREVENT Score		11 (2.6)	18 (4.2)	18 (4.2)	26 (6.1)	19 (4.5)	41 (9.6)	60 (14.1)	69 (16.3)	89 (20.9)	149 (35.1)

## Discussion

This study demonstrates that data available from electronic health record systems can be used to describe the risk of mortality, recurrent stroke, or the combined endpoint of recurrent stroke or death among patients with TIA. The PREVENT score provided robust risk stratification for mortality outcomes (c-statistics of 0.814–0.847) and adequate risk stratification for stroke or death (c-statistics of 0.699–0.744), but modest risk stratification for recurrent stroke (c-statistics of 0.665–0.653). A meta-analysis of studies evaluating the ABCD_2_ score reported an overall c-statistic of 0.72 (0.63 to 0.80) with better performance in the first 7-days post-TIA versus the 90-days post-TIA [16]. These results suggest that TIA risk stratification tools can be implemented within electronic health record systems.

The Learning Healthcare System model has enjoyed widespread adoption as the potential to leverage health data infrastructure to improve care has been recognized [17, 18]. Several approaches to learning from data are consistent with the Learning Healthcare System paradigm. For example, some systems use electronic health record data to develop and report e-quality metrics, which may or may not be reported with risk adjustment [19, 20]. Other systems examine observed outcomes relative to risk-adjusted expected rates [21, 22]. Validated disease specific (e.g., patients with TIA) or setting specific (e.g., patients admitted to the intensive care unit) risk-adjustment models are needed to identify outlier facilities with either lower-than-expected outcome rates or worse-than-expected outcome rates. The identification of outliers is a commonly used approach in quality management [23]. The results of the current study can be used by health systems seeking to evaluate TIA risk-adjusted patient outcomes across facilities.

Our findings may surprise clinicians who might hypothesize that the characteristics of the TIA event (e.g., symptoms) would contribute to risk of recurrent vascular events and hence that approaches which rely solely upon electronic health record data would be inadequate for risk stratification. However, two potential factors may explain the strong performance of the PREVENT score for TIA risk stratification. First, the VA electronic health record includes a broad range of factors including demographics (e.g., age), diagnosis codes (e.g., history of diabetes), medications, laboratory data, and vital signs (e.g., blood pressure). In this way, three of the five ABCD_2_ score elements were included in the PREVENT score. In addition, the inclusion of hospice or palliative care services in the PREVENT score likely contributed to the modeling of mortality. Second, it may be that patients with transient neurological symptoms who have an infarct on brain imaging are coded as a stroke (not a TIA) [24]. Therefore, the value of including brain imaging results to TIA risk stratification must be evaluated within contemporary cohorts with increased use of magnetic resonance imaging [25].

A key characteristic of the PREVENT system was the ability to identify low-risk patients with 0% mortality and 1.4% stroke rates at 90-days. Although all TIA patients should receive timely, guideline-concordant care, such low-risk patients may not require in-patient admission. Future studies should examine whether high-risk patients with 10.8% mortality, 6.7% stroke, and 14.4% stroke or death rates at 90-days could benefit from intensive clinical management approaches [24]. Quality improvement activities may well differ when targeting high-risk versus low-risk patients. For example, in settings with a relatively higher prevalence of high-risk patients, quality improvement programs might seek to promote hospital admission to facilitate timely risk factor management [24].

Given the evidence that outcome rates are improving among TIA patients over time, it is relevant to examine how risk stratification systems perform in contemporary cohorts [1]. A meta-analysis of 40 studies from 2008 to 2015 period reported a cumulative risk of stroke of 7.4% (95% CI 0.043–0.124) at 90 days [1]. The observed 90-day stroke rate of 3.2% in our cohort is lower than these prior reported rates. However, given that we only included recurrent events from VA data sources, we expect that our observed combined endpoint underestimated the actual recurrent stroke events.

Although TIA events are by their nature transient and hence unlikely to confer direct mortality risk, TIAs are markers of vascular disease, [26] and patients with TIA are at increased risk of death [27, 28]. For example, Amarenco, et al. reported that among 4789 patients with TIA or minor stroke treated in specialized centers, major cardiovascular events were observed in 6.2% of patients at 1-year post-index event and death from any cause was observed in 1.8 % [28]. Our finding of 1-year mortality of 7.1% was much higher than the reports from other cohorts.

Although this study included a relatively large sample size from a national cohort of patients with TIA and included a methodologically rigorous design with separate development and validation samples, several limitations merit description. First, the index TIA events were based on diagnosis codes and given the clinical uncertainty inherent in the TIA diagnosis, some of the TIA patients may eventually have been diagnosed with other clinical conditions (e.g., migraine). Second, the electronic health record data did not include symptom characteristics, therefore, we could not compare the CAN or PREVENT scores with the ABCD_2_ score. Future studies should compare the PREVENT score with other clinical TIA risk stratification systems such as the ABCD_2_ score. Third, the cohort included Veteran patients seeking care for an index TIA in a VA hospital. Given differences between the general US population and the Veteran population, the outcome rates may not be generalizable to community cohorts. Future studies should evaluate the performance of the risk models in non-Veteran cohorts. Fourth, the PREVENT score included hospice and palliative care services which may have improved its ability to model mortality outcomes. Future studies should explore how individual components of the score influence the overall stratification ability (e.g., with versus without including hospice patients). Also, given that the VA has a robust electronic health record system, future studies should also examine the implementation of the risk scores in other health systems.

## Conclusions

Although the CAN score and the PREVENT score were similar, the PREVENT score had higher c-statistics for each model, produced greater spread between zenith and nadir risk categories, and contained fewer variables and therefore would be easier to implement. Given that the PREVENT score identified both very low-risk and high-risk TIA populations, Learning Healthcare Systems should consider implementing risk scores using electronic health record data to guide quality management.

## Data Availability

The dataset supporting the conclusions of this article is not available. According to Department of Veterans Affairs (VA) policy, these data are stored behind the VA firewall and cannot be shared even after deidentification. Investigators interested in analyses of the existing data are encouraged to contact the corresponding author.

## Abbreviations

CAN: Clinical Assessment Needs
ED: Emergency Department
PREVENT: Protocol-guided Rapid Evaluation of Veterans Experiencing New Transient Neurological Symptoms
TIA: Transient ischemic attack
VA: Department of Veterans Affairs
PACT: Patient Aligned Care Team
